# Contrasting Regulation of NO and ROS in Potato Defense-Associated Metabolism in Response to Pathogens of Different Lifestyles

**DOI:** 10.1371/journal.pone.0163546

**Published:** 2016-10-03

**Authors:** Jolanta Floryszak-Wieczorek, Magdalena Arasimowicz-Jelonek

**Affiliations:** 1 Department of Plant Physiology, Poznan University of Life Sciences, Wolynska 35, 60–637 Poznan, Poland; 2 Department of Plant Ecophysiology, Faculty of Biology, Adam Mickiewicz University, Umultowska 89, 61–614 Poznan, Poland; Fujian Agriculture and Forestry University, CHINA

## Abstract

Our research provides new insights into how the low and steady-state levels of nitric oxide (NO) and reactive oxygen species (ROS) in potato leaves are altered after the challenge with the hemibiotroph *Phytophthora infestans* or the necrotroph *Botrytis cinerea*, with the subsequent rapid and invader-dependent modification of defense responses with opposite effects. Mainly in the avirulent (avr) *P*. *infestans*–potato system, NO well balanced with the superoxide level was tuned with a battery of SA-dependent defense genes, leading to the establishment of the hypersensitive response (HR) successfully arresting the pathogen. Relatively high levels of S-nitrosoglutathione and S-nitrosothiols concentrated in the main vein of potato leaves indicated the mobile function of these compounds as a reservoir of NO bioactivity. In contrast, low-level production of NO and ROS during virulent (vr) *P*. *infestans*-potato interactions might be crucial in the delayed up-regulation of PR-1 and PR-3 genes and compromised resistance to the hemibiotrophic pathogen. In turn, *B*. *cinerea* triggered huge NO overproduction and governed inhibition of superoxide production by blunting NADPH oxidase. Nevertheless, a relatively high level of H_2_O_2_ was found owing to the germin-like activity in cooperation with NO-mediated HR-like cell death in potato genotypes favorable to the necrotrophic pathogen. Moreover, *B*. *cinerea* not only provoked cell death, but also modulated the host redox milieu by boosting protein nitration, which attenuated SA production but not SA-dependent defense gene expression. Finally, based on obtained data the organismal cost of having machinery for HR in plant resistance to biotrophs is also discussed, while emphasizing new efforts to identify other components of the NO/ROS cell death pathway and improve plant protection against pathogens of different lifestyles.

## Introduction

Generally, both plants and pathogens of different lifestyles have adopted sophisticated strategies to secure their survival and reproduction. Current knowledge and understanding of the ecological and organismal risk of having the machinery for a hypersensitive response (HR) have shown that necrotrophic pathogens impart an ecological cost on plant resistance to biotrophic pathogens. Studies on ecological costs, defined as resulting from any plant mechanisms that provide resistance to biotrophs or hemibiotrophs, but increasing their susceptibility to necrotrophs, are based on the concept that the plant immune system possesses an ability to self-regulate programmed cell death with reference to a “threshold for the hypersensitive response” [1]. In view of the observation by the cited authors, if the environment is abundant in biotrophic pathogens, plants may be subjected to evolutionary pressure to activate a hypersensitive response at a lower threshold. In contrast, necrotrophs dominant in an environment kill host cells using toxic compounds by overcoming the threshold for the initiation of the hypersensitive response, thus facilitating infection. Evidence has demonstrated that colonization of plants by necrotrophic *B*. *cinerea* may largely be prevented by overexpression of anti-apoptotic genes from animals [2]. Moreover, it has been found that the host defense system against a biotroph or hemibiotroph attack might be targeted by toxins of necrotrophic pathogens to enhance virulence and provide susceptibility [3].

Benefits or risks to plants, connected with having the HR mechanism, are frequently associated with a complex of induced compounds and the signaling network that involve many factors affecting stimulation or repression of defense responses against a diverse set of intruders. In the light of these facts, it is crucial to determine factors controlling and modulating a hypersensitive response in host cells [4–7]. Oxidative burst caused by reactive oxygen species (ROS) is the first strong candidate playing an important role in the rapid activation of the genetic program for cellular suicide under different stress conditions [8]. However, most evidence shows that defense responses via ROS, effective against biotrophic pathogens, increase susceptibility to necrotrophic microorganisms (e.g., [1, 9]). Data supporting this concept showed that gray mold development caused by *B*. *cinerea* may be suppressed by spraying the plant with an antioxidant [10, 11].

Especially the necrotrophic fungi kill the host tissue via the secretion of a battery of toxins and degradative enzymes. It was found that secretion of oxalic acid by necrotrophs is the main reason for enormous disturbances in the redox status, which correlated with oxalate-induced cell death in plant tissue [12]. In view of the devastating effects of oxalate-secreting *B*. *cinerea*, host plants have evolved mechanisms of removing apoplastic oxalate and producing H_2_O_2_ by expressing the enzyme oxalate oxidase [13], also known as germin-like proteins (GLPs). Germin-like gene expression leading to increased resistance to *Sclerotinia* spp. has been found in rape [14, 15], peanut [16], sunflower [17], tomato [18] and rice [19]. Because GLPs independent of the NADPH oxidases exhibit superoxide dismutase activity [20], they are probably also involved in R-gene mediated and basal resistance to pathogens of different lifestyles.

Nitric oxide (NO) is the next essential signaling molecule in plant immunity generated as an NO burst, occurring rapidly after pathogen recognition in synergy with ROS (e.g., [21–23]). However, a greater number of investigations focusing on gene expression relevant to the HR have suggested that NO and H_2_O_2_ effects may overlap to a great extent [24], while some observations on the barley response to *Blumeria graminis* indicated that the NO burst preceded H_2_O_2_ synthesis [25]. Pharmacological, biochemical and genetic approaches provide evidence that early overproduction of NO after challenge by a pathogen or an elicitor functions as a messenger in gene-for-gene defense responses and as a general key factor associated with basal resistance in various plant-biotrophic systems (e.g., [26, 27]). Still, much less is known about the role played by NO in the cross-talk between the plant and the necrotrophic pathogen [28–30].

So far, advances in our understanding of plant defense-signaling pathways have shown that plants are capable of recognizing the type of invaders, and they tightly regulate complex defense mechanisms in order to fend off pathogens with various offensive strategies. Both jasmonic acid (JA) and ethylene (ET) have been identified as crucial signaling molecules, effective in triggering plant responses against pathogens, which require host cell death to feed on the remains. In the case of biotrophs and hemibiotrophs, salicylic acid (SA) signaling probably results in resistance [5, 31–33]. Apart from the opposite effects, there is evidence of extensive cross-talk between SA and JA/ET-dependent signaling pathways, which allow the host plant to maximize repression of the spreading invader and establish resistance not only to a single pathogen, but also a simultaneous invasion by biotrophic and necrotrophic forms [27, 34]. Although this idea is generally true and of greater explanatory value, further evidence suggests a more puzzling picture of these interactions [35]. In the environment containing mainly biotrophic pathogens, the salicylic signaling pathway would be preferable over the JA/ET signaling pathway, and such antagonistic effects may trigger HR-like cell death, thereby facilitating subsequent tissue colonization by necrotrophs [1].

One of the many issues which need to be answered is whether there is only one comprehensive program of boosted NO or superoxide formation evoking positive defense responses against pathogens of different lifestyles with respect to the same plant genotype. Since physiological effects of these reactive compounds are related to their concentration, probably synthesis of reactive nitrogen species (RNS) and ROS may be reprogrammed in an invader-dependent manner to compromise plant resistance. However, in contrast to ROS, our understanding of how NO participates in controlling host cell death remains rudimentary.

Therefore our interest was focused on the analysis of the intensity and kinetics of NO burst and assessment of the importance of NO in triggering defense responses in potato genotypes challenged by a hemibiotrophic (*Phytophthora infestans*) or a necrotrophic (*Botrytis cinerea*) pathogen. We attempted to elucidate how RNS have been linked to ROS generation, SA and ethylene signaling compound sensitization, NO-mediated defense responses, e.g. through protein S-nitrosylation or nitration, and PR gene expression leading to HR and/or the collapse of host resistance.

## Materials and Methods

### Plant growth

Sterile potato plants (*Solanum tuberosum* L.) of cv. Bintje–(lacking R genes) highly susceptible to isolate 1.3.4.7.10.11. *Phytophthora infestans* and cv. Bzura–(carrying the *R1* gene [36] and the *R2-like* gene located in potato chromosome IV [37]) highly resistant to 1.3.4.7.10.11. *P*. *infestans* were used in the experiments. Potato plants from *in vitro* tissue culture were transferred to soil and they were grown in a growth chamber with 16 h of light (180 μmol·m^-2^·s^-1^) at 18 ± 2°C and 60% humidity for 4 weeks.

### Pathogen culture

*Botrytis cinerea* Pers.- isolate 1072 (The Bank of Plant Pathogens, Poznań, Poland) was cultured in the dark at a temperature of 23 ± 2°C, on a potato-agar medium pH 6.3 with a 2% addition of glucose. Fungus culture was restored monthly by mycelium passage onto fresh medium.

*Phytophthora infestans—*an isolate 1.3.4.7.10.11. was kindly obtained from the Plant Breeding and Acclimatization Institute, Research Division at Młochów, Poland. The oomycete was grown on a cereal-potato medium with an addition of dextrose.

### Method of inoculation

For *P*. *infestans* inoculation, detached compound leaves of both cultivars were sprayed with a zoospore suspension in water (conc. 2.0 × 10^5^ per ml) on the abaxial leaf surface and kept at 100% humidity in a growth chamber.

For *B*. *cinerea* inoculation, potato compound leaves were sprayed with a conidial suspension (conc. 2.0 × 10^5^ per ml), after which leaves were transferred to the growth chamber at 100% humidity.

The material for further analysis was taken directly or 0.5 h after inoculation (due to technical limitation).

### NO quantification by electrochemical method

All electrochemical measurements were performed by using an universal electrochemical analyser PGSTAT 30 (EcoChemie, Utrecht, the Netherlands). NO generation in leaf tissue was monitored by differential pulse amperometry with an NO-selective needle-type electrode. The electrode was prepared by electropolymerizing a thin film of poly-eugenol on a cleaned Pt needle. This was done by repetitive scanning of the electrode potential between -0.2 and 0.6 V in 10 mM solution of eugenol (Fluka) in 0.1 M NaOH. The modified electrode was then conditioned by applying a constant potential of 0.9 V in a phosphate buffer (pH 7.4) until a stable constant background current was reached. Electrochemical monitoring of NO generation in leaf tissue after pathogen inoculation was performed as described earlier [29]. The current was recalculated into concentration units on the basis of a calibration curve.

### TUNEL assay

The TUNEL assay measures DNA fragmentation using the terminal deoxynucleotidyl transferase (TdT)-mediated deoxyuridine triphosphate (dUTP) nick end labelling method, which involves the TdT-mediated addition of fluorescein-12-dUTP to the 3′-OH ends of fragmented DNA (TUNEL fluorescein; Roche, Indianapolis, IN, USA). Thin slices of the surface of leaf tissue were removed with a bistoury and immediately immersed for 1 h in 4% formaldehyde in phosphate buffered saline (PBS). After being rinsed in PBS, the samples were treated with liquid nitrogen. The samples were rehydrated and incubated for 2 min in Triton X-100 solution (0.1% Triton X-100 in 0.1% sodium citrate) on ice. After rinsing the samples in PBS, the TUNEL reaction was performed according to the manufacturer’s protocol (Roche, http://www.roche.com). Negative controls were conducted in the absence of the TUNEL enzyme. In positive controls, tissue was incubated with DNase I (Roche) for 10 min at 25°C, prior to labelling. The samples were examined using a fluorescence microscope (Axiostar plus, Carl Zeiss, Jena, Germany) equipped with a digital camera, with excitation at 488 nm and emission at 515 nm. Experiments were repeated at least four times with ten slices per treatment. A region of 100 epidermal cells from at least 5 randomly selected slides in each treatment was counted and statistically analysed (results not presented).

### Measurement of ROS

The level of O_2_^•−^ was assayed spectrophotometrically based on the capacity of the superoxide anion-radical to reduce NBT to diformazan [38]. The concentration of H_2_O_2_ was assayed spectrophotometrically using the titanium (Ti^4+^) method [39].

### Detection of peroxynitrite

The level of peroxynitrite was assayed using folic acid as the peroxynitrite scavenger, giving high fluorescent emission products [40]. Leaf discs (1 g) were immersed in the incubation mixture, containing barbital buffer solution (pH 9.4) and folic acid (1.0 x 10^−5^ mol L^-1^). Fluorescence intensity of the solution was recorded at 460 nm with the excitation wavelength set at 380 nm. The standard curve was prepared for 3-Morpholinosydnonimine (SIN-1) from Calbiochem as a donor of peroxynitrite at the range of concentration from 1 to 14 nM.

### NADPH oxidase and germin-like oxidase activities assay

The NADPH dependent O_2_^•−^ generating activity was determined by a modified assay based on a reduction of XTT by O_2_^•−^ anions [41]. Fresh leaves (0.5 g) were homogenized in 50 mM potassium-phosphate buffer, pH 7.0 (1:4; w/v), containing 0.1% Triton X-100 (v/v), 1% PVP, 0.04% Na_2_S_2_O_5_, 1 mM EDTA and centrifuged at 18,000 × g for 20 min. Supernatants were passed through Sephadex G-25 gel filtration columns (Illustra NAP-10, GE Healthcare) and served as the enzyme extract. The volume of 1 ml assay reaction mixture contained 0.5 mM XTT, 0.1 mM NADPH and 30 μl enzyme extract in 50 mM Tris–HCl buffer, pH 7.5. XTT reduction was determined at 470 nm and rates of O_2_^•−^ generation were calculated using an extinction coefficient for XTT of 2.16 × 104 M^−1^ cm^−1^ and the enzyme activity was expressed as O_2_^•−^ per 1 min per 1 mg protein.

Germin-like oxidase activity was assayed using the method as described [42]. Fresh leaves (0.5 g) were homogenized with cold distilled water in 1:3 ratio (w/v) and centrifuged at 15,000 × *g* for 30 min. The reaction mixture contained 0,05 M sodium succinate buffer (pH 4.0), 10 mM CuSO_4_ and enzyme extract (100 μl). The reaction was started by adding 100 mM oxalate. After incubating at 37°C for 10 min, the color reagent (50 mg 4-aminophenazone, 100 mg solid phenol and 1 mg horseradish peroxidase per 100 ml of 0,4 M sodium phosphate buffer, pH 7.0) was added and incubated at room temperature for 15 min. Amount of H_2_O_2_ generated in the assay was calculated using standard curve at 520 nm. One unit of enzyme was defined as the amount of enzyme required to catalyze the generation of 1 nmol of H_2_O_2_ from oxalate per 1 min per 1 mg protein.

### Quantification of total SNOs

Total SNO content was determined by chemiluminescence using a Sievers® Nitric Oxide Analyzer NOA 280i (GE Analytical Instruments, Boulder, CO, USA) according to the procedure proposed earlier [43, 44].

### Immunohistochemical studies of SNOs, GSNO and nitrotyrosine

Detection of SNOs by fluorescence microscopy was performed using the fluorescent Alexa Fluor 405 Hg–Link reagent phenylmercury [45] with some modifications. Potato leaf segments were incubated at 25°C for 1h in darkness with 10 mM NEM prepared in ethanol, and then were washed three times in 10mM Tris–HCl buffer, pH 7.4, for 15 min each. Then they were incubated in the dark with 10μM Alexa Fluor 405 Hg-link phenylmercury (Molecular Probes, USA) for 1h at 25°C. After being washed three times in 10mM Tris–HCl buffer the sections were analyzed with an epifluorescence microscope (Axiostar plus, Zeiss) equipped with an AxioCam MRc 5 camera (Zeiss) using standard filters for Alexa Fluor 405 blue fluorescence (excitation 401nm; emission 421nm). For background staining control sections were incubated with β-mercaptoethanol plus Alexa Fluor 405 and without NEM.

Immunohistochemical localization of GSNO and 3-nitrotyrosine was carried out by fluorescence microscopy of immunofluorescence-stained potato leaf segments using an anti-GSNO rat polyclonal antibody (1:1500) and a rabbit polyclonal antibody against nitrotyrosine (1:300), respectively.

### Ethylene production assay

Leaf discs (diameter = 1.0 cm) were mock inoculated (control) or inoculated with pathogens as described earlier. After incubation, 10 leaf discs were placed in a 20 ml glass vial closed with a crimp top cap with a Teflon/silicon septum. Ethylene was extracted by the SPME (solid phase microextraction) technique with Carboxen/PDMS SPME fibre (Supelco, Bellefonte, PA, USA). The vial septum was pierced with an SPME needle and the fibre was exposed to the headspace over analysed leaves for 10 min. After that time the fibre was retracted into the SPME needle and transferred to the GC injection port, where it was exposed to desorbed volatiles adsorbed on the SPME fibre. The desorption process lasted 10 min, and after that time the fibre was used to sample the next vial. Sampling was performed at 20°C. The gas chromatograph (GC) used for analyses was a Hewlett Packard HP6890 equipped with a split/splitless injector, a flame ionization detector, and a PLOT Al_2_O_3_ KCl (50 m × 0.32 mm × 8 μm) column (19091P-M15, Agilent Technologies, Palo Alto, CA, USA). Helium was used as a carrier gas at a constant pressure of 20 psi. To facilitate sample comparison, amounts of emitted ethylene expressed in nl per 1h per 1 g of FW. All samples were run in triplicate.

### Determination of salicylic acid level

Measurement of SA content essentially followed the protocol described earlier [46]. HPLC analysis was performed on the Agilent HP 1100 HPLC system, equipped with a photodiode array detector (DAD). For all separations a Lichrospher 100 RP18 column (250.0×4.0 mm, 5.0 μm) from Merck was used. The mobile phase consisted of water, methanol, acetic acid (69:28:3, v/v/v), applied in the isocratic elution. The flow rate was adjusted to 1.0 ml/min, the detection wavelength set to DAD at λ = 302.0 nm and 40.0 μl of samples was injected. All separations were performed at the temperature of 45°C. Peaks were assigned by spiking the samples with the standard compound and a comparison of the UV-spectra and retention times (t_R_ = 8 min). The analytical method was validated according to ICH guidelines. The relationship between the peak area and the concentration of salicylic acid injected was found to be highly linear with a regression coefficient *R*^*2*^0.9999. Intra-day repeatability of the HPLC-DAD method was evaluated by performing five repetitive analyses of the standard, which gave an RSD 2 of 15%, showing good precision.

### Defense gene expression

RNA was isolated from 0.2 g of frozen leaf tissues using TriReagent (Sigma). The obtained RNA was purified with the use of a Deoxyribonuclease Kit (Sigma). For the reverse transcription 1 μg of RNA from every experimental variant was processed with a Reverse Transcription Kit (Thermo Scientific Fermentas) according to the manufacturer’s instructions. Real-time PCR was performed on a RotorGene 6000 Thermocycler. The reaction mixture contained 0.1 μM of each primer, 1 μl of 5 x diluted cDNA, 10 μl of the Power SYBR Green PCR Master mix (Applied Biosystems) and DEPC treated water to the total volume of 20 μl. The real-time PCR reaction conditions included an initial 5-minute denaturation at 95°C, followed by 55 cycles consisting of 10 s at 95°C, 20 s at 55°C and 30 s at 72°C. The reaction was finalized by denaturation at a temperature rising from 72°C to 95°C by 1°/5 seconds. Reaction specificity was confirmed by the occurrence of one peak in the melting curve analysis. The data were normalized to two reference genes encoding the elongation factor (ef1α, AB061263) and 18S RNA (X67238). All used primers are presented in Table 1. The Ct values were determined with the use of a Real-time PCR Miner [47] and the relative gene expression was calculated with the use of efficiency corrected calculation models [48].

**Table 1:** Primers used in real-time PCR reaction.

Gene accession number in NCBI database	Gene	Primers
NM_001247429.1	*PR-1*	Forward: GAGCTGGGGACTGCAGGATGC
		Reverse: CCGCGTTGAGCTGGGGGAAA
AF043248.1	*PR-3*	Forward: ACTGGAGGATGGGCTTCAGCA
		Reverse: TGGATGGGGCCTCGTCCGAA
AJ009932.1	*PR-2*	Forward: TTGGCCTTCTGAGGGACACCC
		Reverse: GTGTTCCAGTCCCTCCTTTCACG
X67238	*18S RNA*	Forward: GGGCATTCGTATTTCATAGTCAGAG
		Reverse: CGGTTCTTGATTAATGAAAACATCCT
AB061263	*ef1α*	Forward: ATTGGAAACGGATATGCTCCA
		Reverse: TCCTTACCTGAACGCCTGTCA

### Assessment of disease index

The area affected by disease symptoms was assessed on potato leaves 7 days after inoculation with *P*. *infestans* or *B*. *cinerea* based on a scale from I to IV [49],which represented the percentage of leaf area covered by disease symptoms (I = 1–9%; II = 10–24%; III = 25–49%; IV = 50–100%).

### Statistical analysis

All results are based on three independent experiments, each with at least three biological replicates. For each experiment, means of the obtained values were calculated along with standard deviations. The analysis of variance was conducted and the least significant differences (LSDs) between means were determined using Tukey’s test at the level of significance α = 0.05.

## Results

### NO generation and transfer of NO bioactivity to potato defense system

**Fig 1:**
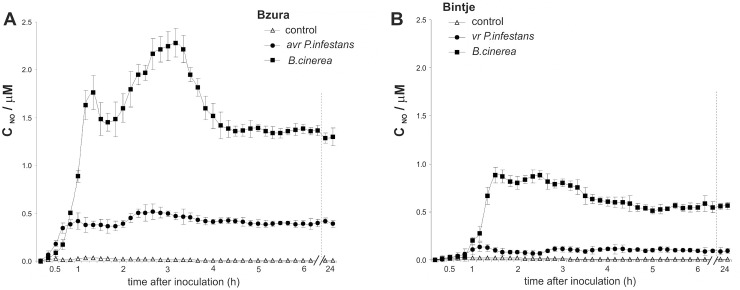
Nitric oxide production in potato-pathogen interaction. Electrochemical detection of NO emission from potato leaves challenged with the hemibiotroph *P*. *infestans* or the necrotroph *B*. *cinerea* in (A) cv. Bzura and (B) Bintje. Values represent the mean ± SD of three independent experiments.

Nitric oxide was generated early, within one hour after challenge with a hemibiotrophic or necrotrophic pathogen in both potato genotypes (Fig 1A and 1B). In the interface between cv. Bzura and *B*. *cinerea* a rapid and high NO burst was observed that reached a maximum (10-fold increase) after 1 hpi of the challenge, and it was followed by a prominent second burst of NO generation (14-fold increase) at 3 hpi in comparison to the mock-inoculated control leaves (Fig 1A). Avr *P*. *infestans* induced the program of boosted NO generation at a considerably lower level (5- to 7-fold increase) than in the necrotroph-dependent NO formation. In turn, an approximately 4-fold rise in NO production was found in the potato Bintje–*B*. *cinerea* interaction, in which it was significantly more abundant when compared to the virulent (vr) *P*. *infestans*–potato response (Fig 1B).

**Fig 2:**
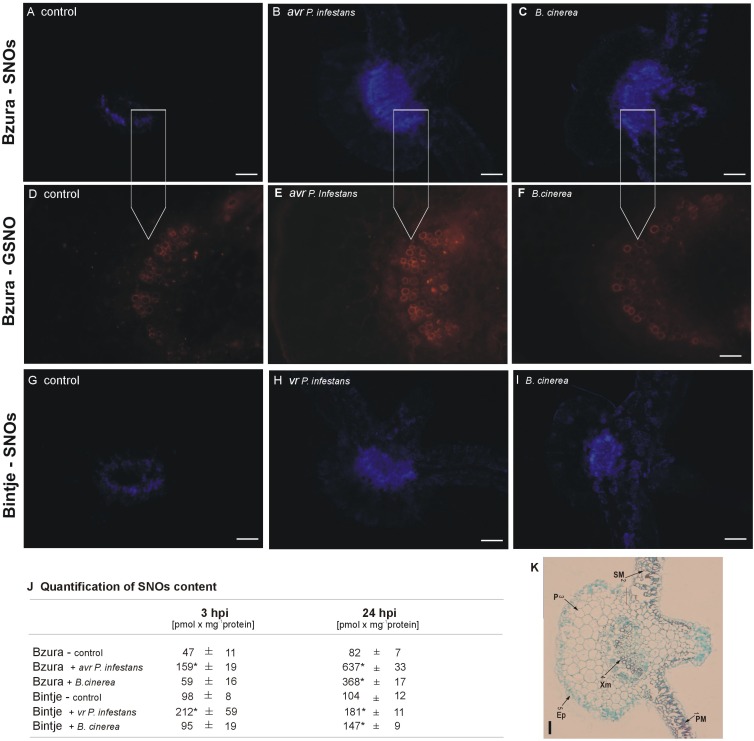
Effect of hemibiotroph (*P*. *infestans)* or necrotroph (*B*. *cinerea*) inoculation on S-nitrosothiols in potato. Total contents of S-nitrosothiols (SNOs) in leaves of potato (A-C) cv. Bzura and (G-I) cv. Bintje inoculated with *P*. *infestans* or *B*. *cinerea* at 48 hpi. Detection of SNOs in potato leaves was performed by immunofluorescence histochemistry using Alexa Fluor 405 Hg–Link reagent phenylmercury; blue fluorescence is attributable to SNOs. Images of leaf cross section show general phenomena representative of three individual experiments. Representative images illustrating the immunodetection of endogenous GSNO in (D) control leaves of potato cv. Bzura and in leaves inoculated with (E) *P*. *infestans* or (F) *B*. *cinerea*. (J) Total SNO content in leaves of potato determined by chemiluminescence assay using Sievers Nitric Oxide Analyzer NOA 280i; (*) significantly different from mock-inoculated potato leaves, P<0.05. Values represent the mean ± SD of at least three independent experiments. (K) Potato leaf cross section viewed under white light: 1 –palisade mesophyll, PM; 2 –spongy mesophyll, SM; 3 –parenchyma, P; 4 –xylem, Xm; 5 –epidermal cells, Ep. Bars indicate 200 μm (A-C, G-I, K) and 100 μm (D-F).

To examine the NO bioactivity transferred to NO-affected proteins we localized total S-nitrosothiols in potato leaves using a fluorescent probe, Alexa Fluor 405 Hg-link (Fig 2A–2C and 2G–2I). The most intensive fluorescence related to the cellular S-nitrosothiol (SNO) pool was observed in the avr *P*. *infestans*–potato interaction, as well as in the *B*. *cinerea*–genotype Bzura interaction at 48 hpi (Fig 2B and 2C). A weak ring of blue fluorescence attributable to SNOs was only observed in the vascular tissue of the petiole in non-infected leaves of cv. Bintje and Bzura (Fig 2A and 2G).

Independently, based on a precise chemiluminescence method, it was found that *P*. *infestans* treatment enhanced the total SNO production approximately 3-fold and more than 7-fold at 3 and 24 hpi, respectively, mainly in leaves of the resistant cv. Bzura. For comparison, a 4.5-fold rise in the SNO pool was observed at 24 hpi in leaves of the same genotype infected by *B*. *cinerea* (Fig 2J). By contrast, no drastic overproduction was recorded in the susceptible genotype Bintje in response to both hemibiotrophic and necrotrophic pathogens. Immunolocalization of S-nitrosoglutathione (GSNO) with a specific antibody also revealed the most abundant orange fluorescence in the vascular and phloem tissues of potato in response to avr *P*. *infestans*, while it was much lower in the Bzura–*B*. *cinerea* interaction (Fig 2D–2F).

**Fig 3:**
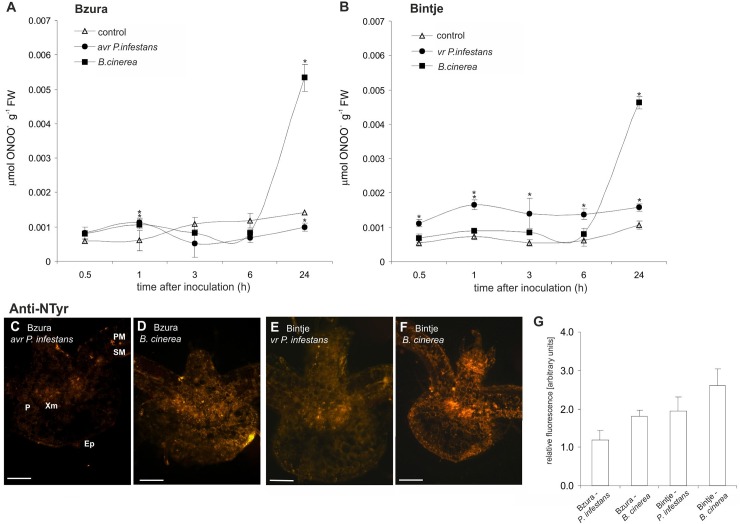
Peroxynitrite formation and tyrosine nitration in potato leaves challenged with *P*. *infestans* or *B*. *cinerea*. Peroxynitrite content in potato challenged with *P*. *infestans* or *B*. *cinerea*, respectively, in (A) cv. Bzura and (B) Bintje. (*) significantly different from mock-inoculated (control) potato leaves, P<0.05. Values represent the mean ± SD of at least three independent experiments. (C-F) Representative images illustrating microscopic localization of protein 3-nitrotyrosine in leaf cross-sections of both potato genotypes challenge inoculated with the pathogens at 24 hpi. Bars indicate 200 μm (C-F). (G) Signal intensities were quantified using Multi Guide V2.2 software (Fujifilm) and expressed in arbitrary units.

Analyses of peroxynitrite formation showed that both potato genotypes challenged with *B*. *cinerea* produced more peroxynitrite than in the case of *P*. *infestans* (Fig 3A and 3B). It was correlated with an immunoassay of nitrated proteins using an antibody against 3-nitrotyrosine (Fig 3C–3F). Intensive orange fluorescence related to highly nitrated proteins was observed in the whole cross-section of petiole and potato leaves infected mainly by *B*. *cinerea* (Fig 3D and 3F) when compared to *P*. *infestans* (Fig 3C and 3E). Similarly, a relative quantification of the microscopic images revealed the highest intensity of orange illumination in potato challenged with *B*. *cinerea*, in contrast to the lowest intensity of fluorescence recorded in the avr *P*. *infestans*–potato system (Fig 3G).

### ROS generation and enzymes involved in their production

**Fig 4:**
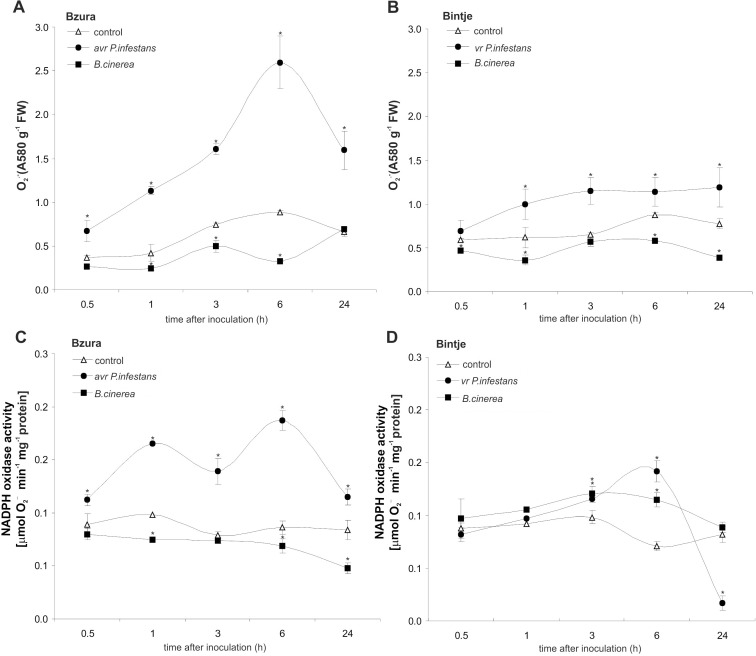
Effect of hemibiotroph *P*. *infestans* or necrotroph *B*. *cinerea* inoculation on superoxide production and NADPH oxidase activity. Superoxide anion generation in potato challenged with *P*. *infestans* or *B*. *cinerea*, respectively, in (A) cv. Bzura and (B) Bintje. NADPH oxidase activity in potato leaves after pathogen inoculation in (C) cv. Bzura and (D) Bintje. (*) significantly different from mock-inoculated (control) potato leaves, P<0.05. Values represent the mean ± SD of at least three independent experiments.

Inoculation of resistant potato with avr *P*. *infestans* caused an early and significant rise in superoxide generation starting from 0.5 hpi with a peak at 6 hpi (5-fold increase) (Fig 4A). In susceptible potato leaves challenged with vr *P*. *infestans* we found a constant 2-fold higher level of the superoxide at the successive time points after pathogen treatment (Fig 4B). In turn, superoxide production was blocked during the first 24 hpi in leaves of both potato genotypes infected with *B*. *cinerea* (Fig 4A and 4B).

To explore whether superoxide managed by NADPH oxidase might be regulated by the NO level, activity of this enzyme was determined in infected potato. Challenge with avr *P*. *infestans* caused an early increase in NADPH oxidase activity starting from 0.5 hpi in resistant potato (Fig 4C). In contrast, *B*. *cinerea* suppressed this enzyme activity during the first day after the necrotroph attack both in the Bzura and Bintje genotypes (Fig 4C and 4D). In turn, treatment of susceptible potato leaves with the virulent *P*. *infestans* showed a weak increase in NADPH oxidase activity only at 6 hpi (Fig 4D).

**Fig 5:**
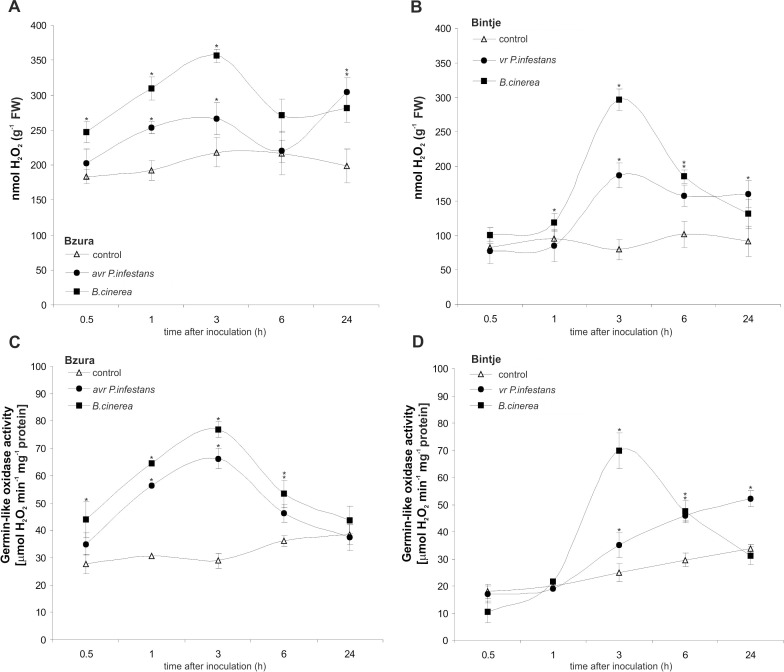
Effect of hemibiotroph *P*. *infestans* or necrotroph *B*. *cinerea* inoculation on hydrogen peroxide production and germin-like oxidase in potato. (A, B) Hydrogen peroxide accumulation and (C, D) germin-like oxidase activity in potato leaves of cv. Bzura and cv. Bintje. (*) significantly different from mock-inoculated (control) potato leaves, P<0.05. Values represent mean ± SD of at least three independent experiments.

Next we investigated whether the total H_2_O_2_ level was also differentially modified by potato exposure to hemibiotroph and necrotroph pathogens. Unexpectedly, a massive rise (3-fold at 3 hpi) of H_2_O_2_ was found in cv. Bzura after challenge with *B*. *cinerea*, and a comparable level was also found in cv. Bintje at the same time after inoculation (Fig 5A and 5B). Likewise, the oomycete *P*. *infestans* induced early H_2_O_2_ synthesis in potato leaves, much more pronounced in the resistant than the susceptible response. Furthermore, the constitutive level of H_2_O_2_ was 2-fold lower in cv. Bintje than in cv. Bzura (Fig 5A and 5B).

When examining the effect of pathogen infection on germin-like activity producing H_2_O_2_ it was found that GLP activity also increased temporarily within 0.5 to 3 hpi after the avr hemibiotroph or necrotroph treatment (Fig 5C). Generally, early induction of GLP activity (at 3 hpi) was higher in the *B*. *cinerea*–potato interaction than in the avr *P*. *infestans*–potato one, and it was more pronounced than in the susceptible responses (Fig 5D).

These data imply that the pathogen-mediated early ROS production is largely dependent on its lifestyle. Shortly after inoculation, the necrotrophic pathogen reduced superoxide production in potato, in contrast to the hemibiotroph. In the resistant potato–*P*. *infestans* interaction, enhanced ROS production was observed via time-dependent activation of NADPH oxidase. Though the necrotroph temporarily blunted NADPH oxidase and superoxide generation, potato leaves restored H_2_O_2_ production owing to germin-like activity.

### Components involved in NO and ROS defense-associated metabolism

**Fig 6:**
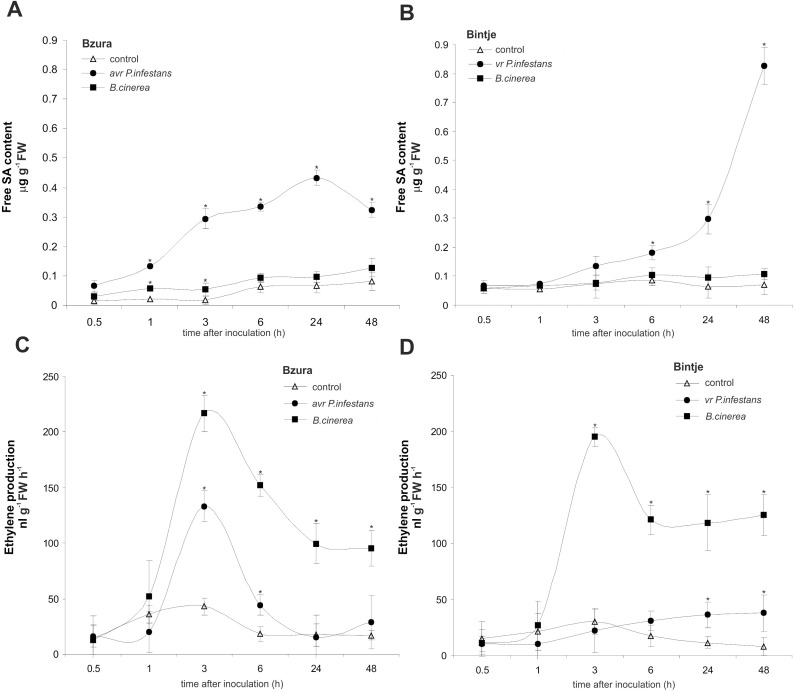
Effect of hemibiotroph *P*. *infestans* or necrotroph *B*. *cinerea* inoculation on free SA level and ethylene production in potato. (A, B) free SA content and (C, D) ethylene production in potato leaves of cv. Bzura and cv. Bintje. (*) significantly different from mock-inoculated (control) potato leaves, P<0.05. Values represent mean ± SD of at least three independent experiments.

To understand how both pathogens change the host redox status and the associated signaling mechanism we next analyzed SA production and SA-dependent defense gene expression. The obtained data indicate that free SA synthesis was mainly triggered by *P*. *infestans* (Fig 6A and 6B). Inoculation with avr *P*. *infestans* induced a systematic rise in the free SA level from 0.5 to 48 hpi (Fig 6A), while the virulent *P*. *infestans* significantly potentiated SA synthesis after 6 hpi (Fig 6B). In contrast, *B*. *cinerea* constantly antagonized SA production in both potato genotypes (Fig 6A and 6B).

Evidence is accumulating that ET together with jasmonic acid plays an important role in the signaling network, creating resistance against necrotrophs; therefore ET production was also detected. *Botrytis cinerea* triggered enhanced ET synthesis peaking at 3 hpi, with similar intensity and kinetics in both potato genotypes (Fig 6C and 6D). The response of susceptible potato to *P*. *infestans* did not reveal ET production (Fig 6D), while the resistant response showed an incidental rise in ET activation only at 3 hpi (Fig 6C).

**Fig 7:**
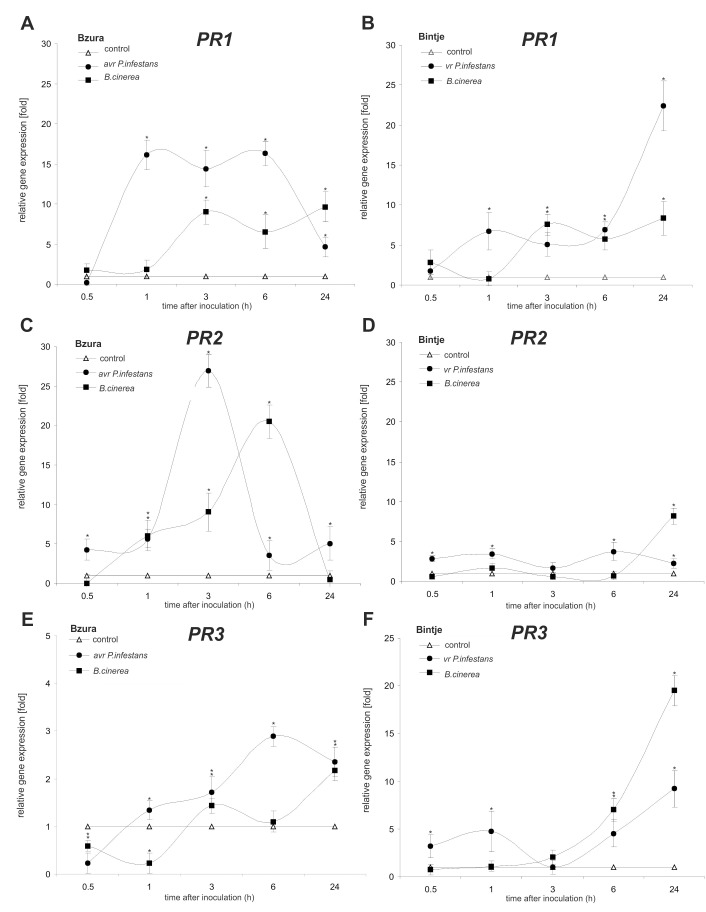
Effect of hemibiotroph *P*. *infestans* or necrotroph *B*. *cinerea* inoculation on *PR* gene expression. (A, B) *PR-1*, (C, D) *PR-2* and (E, F) *PR-3* gene expression in potato leaves of cv. Bzura and cv. Bintje. (*) significantly different from mock-inoculated (control) potato leaves, P<0.05. Values represent mean ± SD of at least three independent experiments.

In order to clarify how redox-based changes induced by pathogens targeted the *PR* genes, we analyzed the amount of mRNA coding for PR-1, PR-2 and PR-3. In the avr *P*. *infestans*–potato interaction the highest amount of mRNA (over 15-fold increase) coding for PR-1 was observed starting from 1–6 hpi, together with increased (over 25-fold) expression of the *PR-2* gene coding for glucanase at 3 hpi (Fig 7A and 7C). Leaves of the susceptible potato cultivar showed strong induction of genes encoding *PR-1* and *PR-3* relatively late (at 24 hpi) after virulent *P*. *infestans* infection (Fig 7B and 7F). In turn, *B*. *cinerea* up-regulated *PR-1* and *PR-2* mainly in potato cv. Bzura (1–3 hpi), but in comparison to the hemibiotrophic pathogen the level of *PR* gene expression was lower and it occurred later after necrotroph inoculation.

### Contrasting functions of induced cell death in potato defense strategy against hemibiotrophic and necrotrophic pathogens

**Fig 8:**
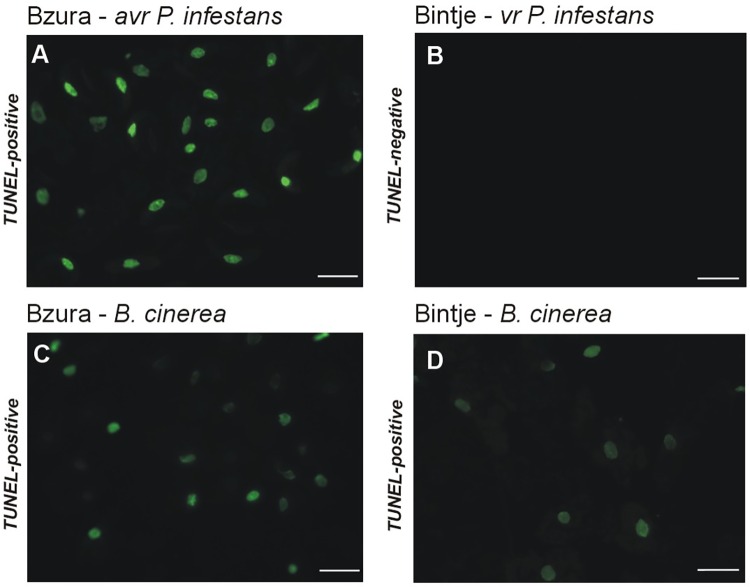
TUNEL test illustrating programmed DNA fragmentation in potato leaves inoculated with the hemibiotroph *P*. *infestans* or with the necrotroph *B*. *cinerea*. (A) TUNEL fluorescein-positive reaction (green nuclei in fluorescence microscopy) detected in the avr *P*. *infestans*–Bzura interaction at 24 hpi; (B) TUNEL fluorescein–negative reaction in *P*. *infestans* susceptible potato cv. Bintje inoculated with the hemibiotroph; TUNEL fluorescein-positive reaction in (C) the Bzura–*B*. *cinerea* and (D) the Bintje–*B*. *cinerea* interactions at 24 hpi. Bars indicate 15 μm (A-D).

High cellular NO and H_2_O_2_ levels in resistant potato attacked by *P*. *infestans* or *B*. *cinerea* positively correlated with symptoms of active cell death, which was identified by the TUNEL-positive assay illustrating the programmed DNA fragmentation (Fig 8A and 8C). After 24 h of the challenge, approximately 95% of potato cells induced by avr *P*. *infestans* contained green-colored nuclei (Fig 8A), while cells elicited by *B*. *cinerea* exhibited only 65% of nuclei with positive TUNEL staining (Fig 8C). In susceptible potato challenged with *B*. *cinerea* very few cells displayed HR-like cell death (Fig 8D), while virulent *P*. *infestans* elicited cell death identified as TUNEL-negative (Fig 8B).

**Fig 9:**
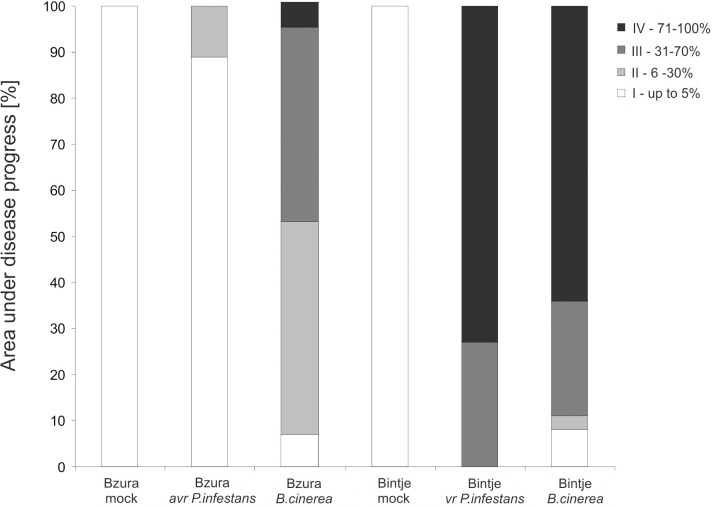
Effect of *P*. *infestans* or *B*. *cinerea* inoculation on disease establishment in susceptible and resistant potato cultivars. The percentage of leaf area infected by the hemibiotroph or necrotroph was measured 7 days after appropriate pathogen challenge inoculation. Values are means from 30 leaves (10 leaves from each of three independent experiments).

Collectively, these findings imply that *B*. *cinerea* triggered HR-like death, which did not arrest the necrotrophic pathogen at the site of its entry, and then these dying cells expanded to form a spreading necrosis. The index of disease assayed 7 days after inoculation showed that *B*. *cinerea* induced development of a strong gray mold covering over 80% and 67% leaf area with disease symptoms in susceptible and resistant potato genotypes, respectively (Fig 9). In the case of avr *P*. *infestans*-inoculated leaves we detected late blight spots covering less than 3% of total leaf area, whereas virulent *P*. *infestans* triggered severe 95% foliar damage observed in each leaf of susceptible potato (Fig 9).

## Discussion

Since the impact of NO on plant metabolism is related to its concentration being precisely regulated by the plant, depending on its abundance NO may provoke opposite effects under physiological and stress conditions [23, 50]. In this study we reported how potato leaves from the same genotypes challenged with pathogens of different lifestyles, e.g. *P*. *infestans* and *B*. *cinerea*, at the sites of attempted infection trigger varying magnitudes of NO burst and subsequent NO/ROS-associated defense signaling linked to an effective hypersensitive response or impaired resistance to the attacker. Our findings show that in contrast to the hemibiotrophic *P*. *infestans*, the necrotrophic *B*. *cinerea* strongly potentiates the NO burst and in synergy with H_2_O_2_ overproduction induces programmed cell death and finally exploits the potato defense system to its own benefit.

It has been well documented by other authors that the necrotrophic *B*. *cinerea* produces non-host specific phytotoxic metabolites, including oxalic acid and botrydial, which by manipulating the host redox milieu favor fungal penetration and colonization of plant tissue [51–53]. In turn, in an avr *P*. *infestans*–potato interaction, boosted NO generation in an induced program was observed to be at a much lower level than the necrotroph-dependent NO formation. Weaker NO-mediated activation was altogether sufficient to culminate in the prompt execution of the HR, found as the TUNEL-positive cell death, which arrested the hemibiotroph at the sites of its attempted entry. At the same time, very low production of NO during vr *P*. *infestans*–potato interactions might be crucial in avoiding active cell death (identified as TUNEL-negative) and could even be an important requirement for pathogen virulence [54].

S-nitrosylation as a redox-based post-translational modification represents the central transfer of NO bioactivity in plants [23, 55]. Considering the augmented NO production detected in infected potato leaves, we next examined whether NO might be converted into SNO storage. The results provided by the chemiluminescent method showed that enhanced NO synthesis found in infected leaves of potato cv. Bzura was accompanied by a significant rise in SNO pools, which was much more pronounced when challenged with *P*. *infestans* rather than *B*. *cinerea*. Relatively high levels of GSNO and SNOs detected immunocytochemically indicated the mobile function of these compounds as a reservoir of NO bioactivity concentrated in the main vein of potato leaves. The tissue-specific location of GSNO was also observed in sunflower hypocotyls exposed to high temperature and mechanical wounding [56, 57]. In our recent paper [44] we reported that in the avr *P*. *infestans*–potato system the NO-coding message was stored and converted into an enhanced total SNO pool and particularly S-nitrosylation of targeted proteins. Among previously identified proteins suggested for S-nitrosylation a reversible and redox-dependent modification of the germin-like protein converting oxalic acid to H_2_O_2_ was found [44, 58]. The proteomic analysis of S-nitrosylated proteins in *Arabidopsis* undergoing a hypersensitive response to *Pseudomonas syringae* pv. *tomato* (*Pst avrB)* also identified a germin-like protein related to its critical role at the onset of the cell death program [59].

Tyrosine nitration as the post-translational modification mediated by peroxynitrite is often considered a marker for certain pathological conditions; however, the nitration process might be involved in the signaling cascade under physiological conditions as well [45]. Probably the excess of ONOO^-^ generated under a pathophysiological state will determine the occurrence of negative modulations translated into an altered protein conformation or catalytic activity of targeted enzymes [60]. In this study, mainly in the ‘Bintje’ genotype treated with the necrotrophic pathogen, we demonstrated boosted tyrosine nitration in proteins to be closely correlated with a late time-dependent rise in the peroxynitrite level and nitro-tyrosine immunoassay. In turn, in an avr *P*. *infestans*-potato system NO was fine-tuned with O_2_^•−^ formed peroxynitrite, probably resetting the signal following pathogen stimulation, resulting in weak ONOO-dependent fluorescence and potato leaf nitration of target proteins. In contrast, a drastic increase in the pool of nitrated proteins, observed in the necrotroph colonized potato tissue, could be considered as a footprint of nitrosative stress [43, 45].

It has been generally accepted that rapid ROS generation is one of the immunity hallmarks in the early host response during an incompatible reaction between the plant and the biotrophic pathogen [4, 61]. In this study inoculation of potato leaves with avr *P*. *infestans* caused early and transient accumulation of O_2_^•−^, reflected in the activation of NADPH oxidase and induction of HR resistance to this biotrophic pathogen. Probably ROS fine-tuned with NO can help the plant to stimulate a particular pathway leading to resistance to the pathogen [62]. Likewise, the results presented earlier [63] indicated that RBOHB silencing in *Nicotiana benthamiana*, in contrast to mutant of NOA1-mediated NO burst, reduced the effective defense against *P*. *infestans*. In *Arabidopsis* with high SNO concentrations NO governed a negative feedback loop compromising HR, mediated by S-nitrosylation of AtRbohD, at Cys 890, blunting its ability to synthesize ROS [64]. Recently it has been reported that although AtRbohD mediates apoplastic ROS production in attacked cells, AtRbohF is crucial for reprogramming plant metabolism and establishing efficient resistance responses in neighboring cells during a susceptible interaction [65].

The present experiments revealed that in contrast to the hemibiotrophic *P*. *infestans*, the necrotrophic *B*. *cinerea* provoked suppression of the superoxide burst and concomitant defense responses in potato leaves. This means that huge amounts of NO, induced mainly by *B*. *cinerea* toxins, might temporarily antagonize the NADPH oxidase dependent O_2_^•−^ production at the preliminary stage of inoculation.

Similarly, recorded data clearly documented that *Sclerotinia sclerotiorum*, a necrotroph related to *B*. *cinerea*, via elicitation of oxalic acid (OA) dampened the host oxidative burst and callose deposition by manipulating the plant redox environment [66]. However, at the later stages when infection was established, this necrotroph induced ROS generation leading to damage of the host tissue. Additionally, tomato leaves inoculated with the non-pathogenic OA-deficient mutant produced oxidative burst and displayed callose deposition [66].

As mentioned before, the host plant provoked by a necrotroph can produce H_2_O_2_ by germin-like proteins and remove apoplastic oxalate [13]. It was found in our study that mainly inoculation with *B*. *cinerea* caused a prompt increase in GLP activity, much greater than avr *P*. *infestans*.

The level of H_2_O_2_ accumulated in bean leaf discs inoculated with different *B*. *cinerea* isolates correlated with their aggressiveness [67]. Our previous experimental approach showed that leaves of ivy pelargonium (*Pelargonium peltatum*) in response to *B*. *cinerea* showed a strong NO burst and H_2_O_2_ accumulation through NO-dependent reversible inhibition of catalase and ascorbate peroxidase [29]. Nitric oxide together with H_2_O_2_ was produced during the initial stages of tissue invasion and also in dying cell layers in *Arabidopsis* mutant genotypes challenged with three pathogenic *Botrytis* species [28].

A growing body of evidence suggests that it is H_2_O_2_ rather than superoxide that synergizes with NO to execute cell death [68]. Accumulation of H_2_O_2_ is known to represent a defense response which is highly effective against biotrophic pathogens [69] but not against necrotrophs [70]. It is well documented that *B*. *cinerea* possesses multiple enzymes that are involved in massive oxidative burst driven by the NADPH oxidase complexes [71] or Cu-Zn superoxide dismutase [11] upon infection in the host plant. Although it is difficult to discriminate between pathogen and plant contribution to ROS generation, this necrotroph potentiates the host oxidative burst and requires ROS-dependent responses to achieve full pathogenicity [72]. The absent of *B*. *cinerea bcsod1* triggered oxidative imbalance (high superoxide and low H_2_O_2_ level) in *Arabidopsis* and tomato plants, reduced *B*. *cinerea* virulence and promoted efficient defense responses [11].

Next, timing and precise control of NO and H_2_O_2_ concentrations are essential in order to orchestrate the programmed cell death with the host resistance to the invader. Experimental data published to date have not been clear whether the cooperation of NO/H_2_O_2_ plays a role in the elicitation of cell death or cell death propagation [24, 73].

Analyses of pathogen-dependent programmed cell death in the Bzura potato genotype showed that *B*. *cinerea* triggered HR-like death, which did not arrest the necrotizing pathogen at the site of its entry, and then these dying cells expanded to form a necrotic region. It is possible that this form of cell death may be the effect of host metabolic dysfunction, in which high NO concentrations play a major role.

Predominantly, the hypersensitive response to a biotroph involves both the early NO burst and SA-dependent signaling, which promotes ROS production [25]. In our experiment despite the high constitutive SA level in potato leaves, a further rise in the free SA level was observed mainly in the potato resistant to avr *P*. *infestans*. In turn, a high NO burst observed after challenging potato leaves with *B*. *cinerea* negatively regulated SA production but not *PR* expression. The existence of additional signals that contribute to the induction of *PR* expression cannot be excluded [74]. As it was revealed, the proton electrochemical gradient across the plasma membrane and sugar metabolism could play a regulatory role in the SA-independent induction of PR gene expression [75, 76].

It also should be taken into account that the induction of a signaling route can activate its own negative feedback, and sometimes this complex network is difficult to interpret. Recent findings in an *Arabidopsis* mutant line (*nox overexpression mutant*) challenged with *P*. *syringae* (PstD3000) also suggested that enhanced NO accumulation compromised multiple modes of disease resistance by blunting both SA synthesis and SA signaling [77]. Also, abscisic acid decreased resistance to *B*. *cinerea* in tomato via reduction of NO, H_2_O_2_ and ET production [78].

We found that both avr *P*. *infestans* and *B*. *cinerea* caused an early ethylene burst in potato leaves, but the magnitude and timing of ET emission substantially differed between these responses. First of all, the *B*. *cinerea*-inducible program of boosted ET synthesis was stimulated early and lasted for the successive hours after inoculation; however, it was not linked with rapid SA-dependent gene up-regulation.

Essentially, *PR-1* gene expression negatively correlates with resistance to *B*. *cinerea*, except for the *Arabidopsis* BOTRYTIS-INDUCED KINASE1 (BIK1) gene mutant highly susceptible to necrotrophic fungal pathogens [79].

Our final data indicate that precise control of early NO production in cooperation with ROS at the site of the pathogen contact with potato leaf tissue activates NO- and ROS-dependent sequences of events for the host’s effective defense against the invader. Mainly in the avr *P*. *infestans*–potato system NO well balanced with the H_2_O_2_ level was tuned with a battery of SA-dependent defense genes, facilitating the establishment of pathogen resistance. The genetic inability of the susceptible potato to accumulate suitable amounts of NO/H_2_O_2_ results in delayed *PR-1* and *PR-3* gene expression and tissue colonization by virulent *P*. *infestans*. Meanwhile, huge NO overproduction in *B*. *cinerea*-challenged potato accelerated HR-like cell death, although it abolished defense-related gene expression and finally plant resistance. These data could indicate that NO is a key player in both PAMP-triggered immunity (PTI) and effector-triggered immunity (ETI) strategies.

The present findings are in line with the concept on the organismal cost of having the machinery for HR as a potential ecological cost of biotroph resistance, causing increased sensitivity to necrotrophic pathogens [1, 80]. It may thus be stated that if plants possess specific signaling equipment to stimulate HR control via NO/H_2_O_2_, they might experience fitness benefits and fitness costs, associated with this defense strategy being overcome by the necrotroph. The necrotrophic *B*. *cinerea*–by manipulating both NO and superoxide synthesis–affects the host redox environment and exploits the potato defense system to its own advantage. Recently the chaperone-like CDC48 was identified as the NO target, and it was suggested that both NO and ROS are probably important components of the mechanism coordinating the ubiquitination system in the cell death pathway [58, 62]. For confirmation, S-nitrosylation of polyubiquitinated proteins was documented by us in potato cells facing a *P*. *infestans* attack [44].

Finally, deep insight into the involvement of NO and ROS closely related to other components and effectors interfering with the potato defense mechanism and inducing side effects might help us apply new environmentally safe methods to improve potato protection against pathogens of different lifestyles.

## References

[1] Kliebenstein DJ, Rowe HC. Ecological costs of biotrophic versus necrotrophic pathogen resistance, the hypersensitive response and signal transduction. Plant Sci. 2008;174: 551–556. 10.1016/j.plantsci.2008.03.005

[2] Dickmann MB, Park YK, Oltersdorf T, Li W, Clemente T, French R. Abrogation of disease development in plants expressing animal antiapoptotic genes. Proc Natl Acad Sci USA 2001;98: 6957–6962. 10.1073/pnas.091108998 11381106 PMC34460

[3] Sweat TA, Wolpert TJ. Thioredoxin h5 is required for victorin sensitivity mediated by a CC-NBS-LRR gene in *Arabidopsis*. Plant Cell 2007;19: 673–687. 10.1105/tpc.106.047563 17322408 PMC1867327

[4] Grant JJ, Loake GJ. Role of reactive oxygen intermediates and cognate redox signaling in disease resistance. Plant Physiol. 2000;124: 21–29. 10.1104/pp.124.1.21 10982418 PMC1539275

[5] Overmyer K, Brosche M, Kangasjarvi J. Reactive oxygen species and hormonal control of cell death. Trends Plant Sci. 2003;8: 335–342. 10.1016/S1360-1385(03)00135-3 12878018

[6] van Breusegem F, Dat JF. Reactive oxygen species in plant cell death. Plant Physiol. 2006;141: 384–390. 10.1104/pp.106.078295 16760492 PMC1475453

[7] Bolwell GP, Daudi A. Reactive oxygen species in plant–pathogen interactions In: del Río LA, Puppo A, editors. Reactive oxygen species in plant signaling, Springer-Verlag Berlin Heidelberg, 2009 pp. 113–133.

[8] Mittler R, Vanderauwera S, Suzuki N, Miller G, Tognetti VB, Vandepoele K, et al ROS signaling: the new wave? Trends Plant Sci. 2011;16: 300–309. 10.1016/j.tplants.2011.03.007 21482172

[9] Govrin EM, Rachmilevitch S, Tiwari BS, Solomon M, Levine A. An elicitor from *Botrytis cinerea* induces the hypersensitive response in Arabidopsis thaliana and other plants and promotes the gray mold disease. Phytopathology 2006;96: 299–307. 10.1094/PHYTO-96-0299 18944445

[10] Elad Y. The use of antioxidants (free radical scavengers) to control grey mould (*Botrytis cinerea*) and white mould (*Sclerotinia sclerotiorum*) in various crops. Plant Pathol. 1992;41: 417–426. 10.1111/j.1365-3059.1992.tb02436.x

[11] López-Cruz J, Crespo-Salvador Ó, Fernández-Crespo E, García-Agustín P, González-Bosch C. Absence of Cu-Zn-superoxide dismutase BCSOD1 reduces *Botrytis cinerea* virulence in *Arabidopsis* and in tomato plants, which reveals interplay among ROS, callose and signaling pathways. Mol Plant Pathol. 2016; 10.1111/mpp.12370 26780422 PMC6638242

[12] Kim KS, Min JY, Dickman MB. Oxalic acid is an elicitor of plant programmed cell death during *Sclerotinia sclerotiorum* disease development. Mol Plant Microbe Interact. 2008;21: 605–612. 10.1094/MPMI-21-5-0605 18393620

[13] Dunwell JM, Khuri S, Gane PJ. Microbial relatives of the seed storage proteins of higher plants: conservation of structure and diversification of function during evolution of the cupin superfamily. Microbiol Mol Biol Rev. 2000;64: 153–179. 10.1128/mmbr.64.1.153-179.2000 10704478 PMC98990

[14] Dong X, Ji R, Guo X, Foster SJ, Chen H, Dong C, et al Expressing a gene encoding wheat oxalate oxidase enhances resistance to *Sclerotinia sclerotiorum* in oil seed rape (*Brassica napus*). Planta 2008;228: 331–340. 10.1007/s00425-008-0740-2 18446363

[15] Rietz S, Bernsdorff FE, Cai D. Members of the germin-like protein family in *Brassica napus* are candidates for the initiation of an oxidative burst that impedes pathogenesis of *Sclerotinia sclerotiorum*. J Exp Bot. 2012;63: 5507–5519. 10.1093/jxb/ers203 22888126 PMC3444267

[16] Livingstone DM, Hampton JL, Phipps PM, Grabau EA. Enhancing resistance to *Sclerotinia minor* in peanut by expressing a barley oxalate oxidase gene. Plant Physiol. 2005;137: 1354–1362. 10.1104/pp.104.057232 15778458 PMC1088326

[17] Hu X. Overexpression of a gene encoding hydrogen peroxide-generating oxalate oxidase evokes defense responses in sunflower. Plant Physiol. 2003;133: 170–181. 10.1104/pp.103.024026 12970484 PMC196595

[18] Walz A, Zingen-Sell I, Loeffler M, Sauer M. Expression of an oxalate oxidase gene in tomato and severity of disease caused by *Botrytis cinerea* and *Sclerotinia sclerotiorum*. Plant Pathol. 2008;57: 453–458. 10.1111/j.1365-3059.2007.01815.x

[19] Manosalva PM, Davidson RM, Liu B, Zhu X, Hulbert SH, Leung H, et al A germin-like protein gene family functions as a complex quantitative trait locus conferring broad-spectrum disease resistance in rice. Plant Physiol. 2009;149: 286–296. 10.1104/pp.108.128348 19011003 PMC2613727

[20] Christensen AB, Thordal-Christensen H, Zimmermann G, Gjetting T, Lyngkjaer MF, Dudler R, et al The germin-like protein GLP4 exhibits superoxide dismutase activity and is an important component of quantitative resistance in wheat and barley. Mol Plant Microbe Interact. 2004;17: 109–117. 10.1094/mpmi.2004.17.1.109 14714874

[21] García-Brugger A, Lamotte O, Vandelle E, Bourque S, Lecourieux D, Poinnsot B, et al Early signaling events induced by elicitors of plant defences. Mol Plant Microbe Interact. 2006;7: 711–724. 10.1094/MPMI-19-0711 16838784

[22] Neil S, Hancock JT, Wilson ID. Reactive oxygen species, nitric oxide and signal crosstalk In: Yoshioka K, Shinozaki K, editors. Signal crosstalk in plant stress responses, Wiley-Blackwell; 2009 pp. 136–160.

[23] Yu M, Lamattina L, Spoel SH, Loake GJ. Nitric oxide function in plant biology: a redox cue in deconvolution. New Phytol. 2014;202: 1142–1156. 10.1111/nph.12739 24611485

[24] Zago E, Morsa S, Dat JF, Alard P, Ferrarini A, Inze D, et al Nitric oxide- and hydrogen peroxide-responsive gene regulation during cell death induction in tobacco. Plant Physiol. 2006;141: 404–411. 10.1104/pp.106.078444 16603664 PMC1475440

[25] Mur LAJ, Kenton P, Lloyd AJ, Ougham H, Prats E. The hypersensitive response; the centenary is upon us but how much do we know? J Exp Bot. 2008;59: 501–520. 10.1093/jxb/erm239 18079135

[26] Zeier J, Delledonne M, Mishina T, Severi E, Sonoda M, Lamb C. Genetic elucidation of nitric oxide signaling in incompatible plant–pathogen interactions. Plant Physiol. 2004;136: 2875–2886. 10.1104/pp.104.042499 15347797 PMC523349

[27] Mur LAJ, Kenton P, Atzorn R, Miersch O, Wasternack C. The outcomes of concentration-specific interactions between salicylate and jasmonate signaling include synergy, antagonism, and oxidative stress leading to cell death. Plant Physiol. 2006;140: 249–262. 10.1104/pp.105.072348 16377744 PMC1326048

[28] van Baarlen P, Woltering EJ, Staats M, van Kan JAL. Histochemical and genetic analysis of host and non-host interactions of *Arabidopsis* with three *Botrytis* species: An important role for cell death control. Mol Plant Pathol. 2007;8: 41–54. 10.1111/j.1364-3703.2006.00367.x 20507477

[29] Floryszak-Wieczorek J, Arasimowicz M, Milczarek G, Jeleń H, Jackowiak H. Only an early nitric oxide burst and the following wave of secondary nitric oxide generation enhanced effective defence responses of pelargonium to a necrotrophic pathogen. New Phytol. 2007;175: 718–730. 10.1111/j.1469-8137.2007.02142.x 17688587

[30] Asai S, Yoshioka H. Nitric oxide as a partner of reactive oxygen species participates in disease resistance to nectrotophic pathogen *Botryis cinerea* in *Nicotiana benthamiana*. Mol Plant Microbe Interact. 2009;22: 619–629. 10.1094/MPMI-22-6-0619 19445587

[31] Asai T, Stone JM, Heard JE, Kovtun Y, Yorgey P, Sheen J, et al Fumonisin B1-induced cell death in *Arabidopsis* protoplasts requires jasmonate-, ethylene-, and salicylate-dependent signaling pathways. Plant Cell. 2000;12: 1823–1835. 10.1105/tpc.12.10.1823 11041879 PMC149122

[32] McDowell JM, Dangl JL. Signal transduction in the plant immune response. Trends Plant Sci. 2000;290: 79–82. 10.1016/s0968-0004(99)01532-7 10664588

[33] Glazebrook J. Contrasting mechanisms of defense against biotrophic and necrotrophic pathogens. Annu Rev Phytopathol. 2005;43: 205–227. 10.1146/annurev.phyto.43.040204.135923 16078883

[34] Halim VA, Altmann S, Ellinger D, Eschen-Lippold L, Miersch O, Scheel D, et al PAMP-induced defense responses in potato require both salicylic acid and jasmonic acid. Plant J. 2009;57: 230–242. 10.1111/j.1365-313X.2008.03688.x 18801014

[35] Vleesschauwer D, Chernin L, Höfte MM. Differential effectiveness of *Serratia plymuthica* IC1270-induced systemic resistance against hemibiotrophic and necrotrophic leaf pathogens in rice. BMC Plant Biol. 2009;9: 9 10.1186/1471-2229-9-9 19161601 PMC2650696

[36] Gebhardt C, Ballvora A, Walkemeier B, Oberhagemann P, Schuler K. Assessing genetic potential in germplasm collections of crop plants by marker-trait association: a case study for potatoes with quantitative variation of resistance to late blight and maturity type. Mol Breed. 2004;13: 93–102. 10.1023/b:molb.0000012878.89855.df

[37] Plich J, Tatarowska B, Lebecka R, Śliwka J, Zimnoch-Guzowska E, Flis B. R2-like gene contributes to resistance to *Phytophthora infestans* in polish potato cultivar Bzura. Am J Potato Res. 2015;92: 350–358 10.1007/s12230-015-9437-9

[38] Doke N. Involvement of superoxide anion generation in the hypersensitive response of potato tuber tissues to infection with an incompatible race *Phytophthora infestans* and to the hyphal wall components. Physiol Plant Pathol. 1983;23: 345–357. 10.1016/0048-4059(83)90019-x

[39] Becana M, Aparicio-Tejo P, Irigoyen JJ, Sanchez-Diaz M. Some enzymes of hydrogen peroxide metabolism in leaves and root nodules of *Medicago sativa*. Plant Physiol. 1986;82: 1169–1171. 10.1104/pp.82.4.1169 16665158 PMC1056282

[40] Huang JC, Li DJ, Diao JC, Hou J, Yuan JL, Zou GL. A novel fluorescent method for determination of peroxynitrite using folic acid as a probe. Talanta 2007;72: 1283–1287. 10.1016/j.talanta.2007.01.033 19071757

[41] Able AJ, Guest DI, Sutherland MW. Use of a new tetrazolium-based assay to study the production of superoxide radicals by tobacco cell cultures challenged with avirulent zoospores of *Phytophthora parasitica* var *nicotianae*. Plant Physiol. 1998;117: 491–499. 10.1104/pp.117.2.491 9625702 PMC34969

[42] Kumar R, Hooda V, Pundir CS. Purification and partial characterization of oxalate oxidase from leaves of forage Sorghum (*Sorghum vulgare* var. KH-105) seedlings. Indian J Biochem Biophys. 2011;48: 42–46. 21469601

[43] Valderrama R, Corpas FJ, Carreras A, Fernandez-Ocana A, Chaki M, Luque F, et al Nitrosative stress in plants. FEBS Lett. 2007;581: 453–461. 10.1016/j.febslet.2007.01.006 17240373

[44] Abramowski D, Arasimowicz-Jelonek M, Izbiańska K, Billert H, Floryszak-Wieczorek J. Nitric oxide modulates redox-mediated defense in potato challenged with *Phytophthora infestans*. Eur J Plant Pathol. 2015;143: 237–260. 10.1007/s10658-015-0677-9

[45] Corpas FJ, Chaki M, Fernández-Ocaña A, Valderrama R, Palma JM, Carreras A, et al Metabolism of reactive nitrogen species in pea plants under abiotic stress conditions. Plant Cell Physiol. 2008;49: 1711–1722. 10.1093/pcp/pcn144 18801763

[46] Meuwly P, Métraux JP. Ortho-anisic acid as internal standard for the simultaneous quantification of salicylic acid and its putative biosynthetic precursors in cucumber leaves. Anal Biochem. 1993;214: 500–505. 10.1006/abio.1993.1529 8109740

[47] Zhao S, Fernald RD. Comprehensive algorithm for quantitative real-time polymerase chain reaction. J Comput Biol. 2005;12: 1047–1064. 10.1089/cmb.2005.12.1047 16241897 PMC2716216

[48] Pfaffl MW. A new mathematical model for relative quantification in real-time RT-PCR. Nucleic Acids Res. 2001;29: e45 10.1093/nar/29.9.e45 11328886 PMC55695

[49] James WC. An illustrated series of assessment keys for plant diseases, their preparation and usage. Can Plant Dis Sur. 1971;51: 39–65.

[50] Chen YH, Shen HL, Hsu PJ, Hwang SG, Cheng WH. N-acetylglucosamine-1-P uridylyltransferase 1 and 2 are required for gametogenesis and embryo development in *Arabidopsis thaliana*. Plant Cell Physiol. 2014;55: 1977–1993. 10.1093/pcp/pcu127 25231969

[51] Cessna SG, Sears VE, Dickman MB, Low PS. () Oxalic acid, a pathogenicity factor for *Sclerotinia sclerotiorum*, suppresses the oxidative burst of the host plant. Plant Cell 2000;12: 2191–2200. 10.2307/3871114 11090218 PMC150167

[52] Colmenares AJ, Aleu J, Duran-Patron R, Collado IG, Hernandez-Galan R. The putative role of botrydial and related metabolites in the infection mechanism of *Botrytis cinerea*. J Chem Ecol. 2002;28: 997–1005. 12049236 10.1023/a:1015209817830

[53] van Kan JAL. Licensed to kill: the lifestyle of a necrotrophic plant pathogen. Trends Plant Sci. 2006;11: 1360–1385. 10.1016/j.tplants.2006.03.005 16616579

[54] Mur LAJ, Kenton P, Draper J. *In planta* measurements of oxidative bursts elicited by avirulent and virulent bacterial pathogens suggests that H_2_O_2_ is insufficient to elicit cell death in tobacco. Plant Cell Environ. 2005;28: 548–561. 10.1111/j.1365-3040.2005.01301.x

[55] Kovacs I, Lindermayr C. Nitric oxide-based protein modification: formation and site-specificity of protein S-nitrosylation. Front Plant Sci. 2013; 4, 137 10.3389/fpls.2013.00137 23717319 PMC3653056

[56] Chaki M. Valderrama R, Fernández-Ocaña AM, Carreras A, Gómez-Rodíguez MV, Pedrajas JR, et al Mechanical wounding induces a nitrosative stress by down-regulation of GSNO reductase and an increase in S-nitrosothiols in sunflower (*Helianthus annuus*) seedlings. J Exp Bot. 2011;62: 1803–1813. 10.1093/jxb/erq358 21172815 PMC3060671

[57] Chaki M, Valderrama R, Fernández-Ocaña AM, Carreras A, Gómez-Rodríguez MV, López-Jaramillo J, et al High temperature triggers the metabolism of S-nitrosothiols in sunflower mediating a process of nitrosative stress which provokes the inhibition of ferredoxin-NADP reductase by tyrosine nitration. Plant Cell Environ. 2011;34: 1803–1818. 10.1111/j.1365-3040.2011.02376.x 21676000

[58] Astier J, Besson-Bard A, Lamotte O, Bertoldo J, Bourque S, Terenzi H, et al Nitric oxide inhibits the ATPase activity of the chaperone-like AAA+ ATPase CDC48, a target for S-nitrosylation in cryptogein signalling in tobacco cells. Biochem J. 2012;447: 249–60. 10.1042/BJ20120257 22835150

[59] Romero-Puertas MC, Campostrini N, Mattè A, Righetti PG, Perazzolli M, Zolla L, et al Proteomic analysis of S-nitrosylated proteins in *Arabidopsis thaliana* undergoing hypersensitive response. Proteomics 2008;8: 1459–1469. 10.1002/pmic.200700536 18297659

[60] Arasimowicz-Jelonek M, Floryszak-Wieczorek J. Understanding the fate of peroxynitrite in plant cells–from physiology to pathophysiology. Phytochemistry 2011;72: 681–688. 10.1016/j.phytochem.2011.02.025 21429536

[61] Shetty NP, Jørgensen HJL, Jensen JD, Collinge DB, Shetty HS. Roles of reactive oxygen species in interactions between plants and pathogens. Eur J Plant Pathol. 2008;121: 267–280. 10.1007/978-1-4020-8780-6_6

[62] Trapet P, Kulik A, Lamotte O, Jeandroz S, Bourque S, Nicolas-Francès V, et al NO signaling in plant immunity: A tale of messengers. Phytochemistry 2015;112: 72–79. 10.1016/j.phytochem.2014.03.015 24713571

[63] Asai S, Ohta K, Yoshiokaa H. MAPK signaling regulates nitric oxide and NADPH oxidase-dependent oxidative bursts in *Nicotiana benthamiana*. Plant Cell 2008;20: 1390–1406. 10.1105/tpc.107.055855 18515503 PMC2438462

[64] Yun BW, Feechan A, Yin M, Saidi NB, Le Bihan T, Yu M, et al S-nitrosylation of NADPH oxidase regulates cell death in plant immunity. Nature 2011;478: 264–268. 10.1038/nature10427 21964330

[65] Chaouch S, Queval G, Noctor G. AtRbohF is a crucial modulator of defence-associated metabolism and a key actor in the interplay between intracellular oxidative stress and pathogenesis responses in *Arabidopsis*. Plant J. 2012;69: 613–627. 10.1111/j.1365-313X.2011.04816.x 21985584

[66] Williams B, Kabbage M, Kim HJ, Britt R, Dickman MB. Tipping the balance: *Sclerotinia sclerotiorum* secreted oxalic acid suppresses host defenses by manipulating the host redox environment. PLoS Pathogens 2011;7: e1002107 10.1371/journal.ppat.1002107 21738471 PMC3128121

[67] Tiedemann AV. Evidence for a primary role of active oxygen species in induction of host cell death during infection of bean leaves with *Botrytis cinerea*. Physiol Mol Plant Pathol. 1997;50: 151–166. 10.1006/pmpp.1996.0076

[68] Delledonne M, Zeier J, Marocco A, Lamb C. Signal interactions between nitric oxide and reactive oxygen intermediates in the plant hypersensitive disease response. Proc Natl Acad Sci USA 2001;98: 13454–13459. 10.1073/pnas.231178298 11606758 PMC60892

[69] Hückelhoven R, Fodor J, Preis C, Kogel KH. Hypersensitive cell death and papilla formation in barley attacked by the powdery mildew fungus are associated with H_2_O_2_ but not with salicylic acid accumulation. Plant Physiol. 1999;119: 1251–1260. 10.1104/pp.119.4.1251 10198083 PMC32009

[70] Horbach R, Navarro-Quesada AR, Knogge W, Deising HB. When and how to kill a plant cell: infection strategies of plant pathogenic fungi. J Plant Physiol. 2011;168: 51–62. 10.1016/j.jplph.2010.06.014 20674079

[71] Siegmund U, Heller J, van Kan JA, Tudzynski P. The NADPH oxidase complexes in Botrytis cinerea: evidence for a close association with the ER and the tetraspanin Pls1. PLoS One 2013;8(2): e55879 10.1371/journal.pone.0055879 23418468 PMC3572182

[72] Heller J, Tudzynsky P. Reactive oxygen species in phytopathogenic fungi: signaling, development and disease. Annu Rev Phytopathol. 2011;49, 369–390. 10.1146/annurev-phyto-072910-095355 21568704

[73] Tada Y, Mori T, Shinogi T, Yao N, Takahashi S, Betsuyaku S, et al Nitric oxide and reactive oxygen species do not elicit hypersensitive cell death but induce apoptosis in the adjacent cells during the defense response of oat. Mol Plant Microbe Interact. 2004;17: 245–253. 10.1094/MPMI.2004.17.3.245 15000391

[74] Van Kaan JAL, Cozijnsen T, Danhash N, De Wit PJGM. Induction of tomato stress protein mRNAs by ethephon, 2,6-dichloroisonicotinic acid and salicylate. Plant Mol Biol 1995;27: 1205–1213. 10.1007/bf00020894 7766902

[75] Schaller A, Roy P, Amrhein N. Salicylic acid-independent induction of pathogenesis-related gene expression by fusicoccin. Planta 2000;210: 599–606. 10.1007/s004250050049 10787053

[76] Campos-Soriano L, San Segundo B. New insights into the signaling pathways controlling defense gene expression in rice roots during the arbuscular mycorrhizal symbiosis. Plant Signal Behav. 2011;6: 553–557. 10.4161/psb.6.4.14914 21422823 PMC3142391

[77] Yun BW, Skelly MJ, Yin M, Yu M, Mun BG, Lee SU, et al Nitric oxide and S-nitrosoglutathione function additively during plant immunity. New Phytol. 2016;211: 516–26. 10.1111/nph.13903 26916092

[78] Sivakumaran A, Akinyemi A, Mandon J, Cristescu SM, Hall MA, Harren FJ, et al ABA Suppresses *Botrytis cinerea* elicited NO production in tomato to influence H_2_O_2_ generation and increase host susceptibility. Front Plant Sci. 2016;25(7): 709 10.3389/fpls.2016.00709 27252724 PMC4879331

[79] Veronese P, Chen X, Bluhm B, Salmeron J, Dietrich RA, Mengiste T. The BOS loci of Arabidopsis are required for resistance to *Botrytis cinerea* infection. Plant J. 2004;40: 558–574. 10.1111/j.1365-313X.2004.02232.x 15500471

[80] Spoel SH, Johnson JS, Dong X. Regulation of tradeoffs between plant defenses against pathogens with different lifestyles. Proc Natl Acad Sci USA 2007;104: 18842–18847. 10.1073/pnas.0708139104 17998535 PMC2141864

